# Comparative analysis of bacterial communities and environmental interactions in seawater and saline-alkali aquaculture ponds for *Scylla paramamosain* in northern China

**DOI:** 10.3389/fmicb.2025.1589304

**Published:** 2025-05-21

**Authors:** Dongping Zhou, Yuanyuan Fu, Lei Liu, Weichuan Lin, Zhibin Lu, Yangfang Ye, Minglei Zhang

**Affiliations:** ^1^School of Marine Sciences, Ningbo University, Ningbo, China; ^2^Southern Ocean Science and Engineering Guangdong Laboratory (Zhanjiang), Zhanjiang, China; ^3^Ningbo Institute of Oceanography, Ningbo, Zhejiang, China; ^4^Shandong Freshwater Fisheries Research Institute, Jinan, China

**Keywords:** *Scylla paramamosain*, bacterial communities, 16S rRNA gene sequencing, aquaculture, seawater pond, saline-alkali water pond

## Abstract

As the aquaculture capacity of *S. paramamosain* in southern China nears saturation, northern coastal regions, which are characterized by abundant water resources, ample feed availability, and favorable climatic conditions, have emerged as ideal areas for aquaculture expansion. This study investigates the aquatic environment of *S. paramamosain* cultured in seawater and saline-alkali ponds in northern China. Over the course of a five-month aquaculture experiment, water samples were collected from seawater and saline-alkali ponds and subsequently analyzed using 16S rRNA gene sequencing technology to examine the bacterial community composition and its relationship with physicochemical water quality parameters. Sensitive bacterial species were identified as well. The results revealed that seawater ponds exhibited higher salinity and dissolved oxygen levels, but lower pH, ammonia nitrogen, and nitrite nitrogen concentrations. In contrast, saline-alkali ponds exhibited elevated pH, ammonia nitrogen, and nitrite nitrogen levels, accompanied by reduced salinity and dissolved oxygen. Bacterial communities in seawater ponds demonstrated greater species richness, evenness, and diversity indices, whereas those in saline-alkali ponds were characterized by reduced diversity and distinct dominant bacterial groups. Redundancy analysis (RDA) identified salinity, pH, and dissolved oxygen as the principal environmental factors influencing bacterial community structure. Using the IndVal method, we identified strong associations between specific bacterial species and pond types, such as *Sphingoaurantiacus* and *Cobetia* in seawater ponds, and *Roseivivax*, *Tropicimonas*, and *Thiobacillus* in saline-alkali ponds. Environmental factors exerted distinct effects on bacterial communities in the two pond types, with sensitive bacterial species demonstrating significant specificity and strong correlations with water quality parameters. Functional predictions indicated that microbes in saline-alkali ponds prioritized resource acquisition and stress resistance, whereas those in seawater ponds emphasized nitrogen metabolism and protein synthesis. This study demonstrated significant differences in bacterial community characteristics between seawater and saline-alkali ponds, which were strongly influenced by water quality parameters. These findings are crucial for optimizing the growth environment of *S. paramamosain*, providing essential data for improving aquaculture conditions and promoting the development of northern *S. paramamosain* farming.

## 1 Introduction

The mud crab (*Scylla paramamosain*) is an important commercial crustacean species distributed throughout the Indo-Pacific region (Lin et al., 2012; Zhang et al., 2024). In China, the distribution and commercial production of *S. paramamosain* are primarily concentrated in the coastal regions south of the Yangtze River estuary (Ma et al., 2014; Zhou et al., 2023). Currently, the aquaculture capacity of *S. paramamosain* in southern China has reached saturation, necessitating the development of additional cultivation areas to meet increasing demand. The northern coastal regions of China are characterized by abundant water resources, ample feed availability, and a suitable climate, making them the preferred areas for the expanded cultivation of *S. paramamosain*. For instance, the Yellow River Delta region (37°35′–38°12’N, 118°33′–119°20′E), a recently formed landmass resulting from sediment deposition carried by the Yellow River (Cui et al., 2009), is a promising candidate for the expansion of *S. paramamosain* aquaculture. However, it is important to note that the Yellow River Delta (YRD) region exhibits distinctive salinization patterns characterized by complex salt compositions and spatial heterogeneity. The predominant salt types include NaCl, Na2SO4, and NaHCO3, with sodium ions constituting 70–85% of exchangeable cations in surface soils (Huang et al., 2015). Soil salinity typically ranges from 0.3 to 3.0%, showing significant seasonal variation due to the monsoon climate – higher in dry seasons (spring) and lower after summer rainfall (Komiyama et al., 2020). These factors may pose challenges to the cultivation of *S. paramamosain* in the region and should be carefully considered in the development of aquaculture practices.

Aquaculture ponds are complex ecosystems in which microorganisms in both water and sediment interact (Lahiri et al., 2018). Microorganisms influence the entire biogeochemical cycle and are crucial to productivity, nutrient cycling, water quality, and the health of farmed animals (Falkowski et al., 2008). Microbial communities are well-known to respond rapidly to environmental changes, such as pH, oxygen concentration, nitrogen concentration, and salinity (Xiong et al., 2015). The close relationship between water quality and bacterial communities in aquaculture ponds has been well-documented. Bacterial communities help maintain the aquatic ecosystem and can serve as indicators of its wellbeing (Paerl et al., 2003). Furthermore, bacteria are an integral part of the diet of various fish and crustaceans in aquaculture environments, including rotifers, copepods, shrimp, and fish (Troch et al., 2012). Bacteria are occasionally discussed as potential sources of essential nutrients and are considered viable food sources within the aquaculture food chain (Nichols, 2003). Clearly, bacteria represent a critical component of pond ecosystems, and the structure and diversity of their communities significantly impact the overall health of these ecosystems (Vincent et al., 2008). However, current studies on bacterial communities in *S. paramamosain* aquaculture ponds are primarily limited to the southern coastal regions of China (Wei et al., 2019; Kasan et al., 2021), with bacterial communities in northern China remaining largely unexplored.

Water temperature and salinity are key physicochemical parameters that significantly influence bacterial community structure and diversity, which in turn affect the growth of *S. paramamosain* (Deng et al., 2020). For example, studies on *Litopenaeus vannamei* aquaculture ponds have shown that temperature fluctuations between 25 and 35°C promote the proliferation of potential pathogenic bacteria, such as *Vibrio* species (Cheng et al., 2005). The excessive growth of *Vibrio* bacteria can degrade water quality by accumulating harmful substances, including ammonia nitrogen and nitrite, and depleting dissolved oxygen levels (Qiu et al., 2023; Zhang et al., 2022). Regarding salinity, deviations from the optimal range—whether excessively high or low—can disrupt bacterial community composition, altering the balance between beneficial and harmful bacteria (Gorrasi et al., 2021; Wang et al., 2019). A decline in beneficial bacteria can destabilize essential ecological processes, such as the nitrogen cycle, thereby impairing the efficient transformation of metabolic byproducts like ammonia nitrogen. This degradation in water quality can hinder the growth and survival of crab species (Hastuti et al., 2021).

The pH is a critical physicochemical parameter that profoundly influences bacterial communities, thereby affecting the growth of *S. paramamosain* (Deng et al., 2020). Studies have shown that in acidic environments with reduced pH, acidophilic bacteria, such as *Thiobacillus* species, tend to dominate the bacterial community (Johnson and Quatrini, 2020). These bacteria produce acidic byproducts during their metabolic processes, further lowering the water’s pH and exacerbating acidification (Harrison, 1984; Crescenzi et al., 2006). Acidified aquatic environments can disrupt the osmoregulation and respiratory metabolism of *S. paramamosain*, resulting in impaired growth, molting difficulties, weakened immunity, and heightened susceptibility to pathogens (Yuan et al., 2024).

Dissolved oxygen (DO) levels are crucial for both bacterial communities and the growth of *S. paramamosain* (Jie et al., 2021). In low-DO environments, facultative anaerobic bacteria, such as *Enterobacter* species, tend to proliferate (Kato et al., 1997). The overgrowth of *Enterobacter* bacteria depletes oxygen and produces toxic byproducts, such as ammonia nitrogen and hydrogen sulfide, which deteriorate water quality (Wang et al., 2019). This decline in water quality can induce hypoxia in *S. paramamosain*, leading to diminished feeding, impaired growth, and even mortality (Wang et al., 2021).

Bacterial communities also influence physicochemical water quality parameters, thereby indirectly affecting the growth environment of *S. paramamosain* (Pati et al., 2023). For example, nitrifying bacteria play a crucial role in the nitrogen cycle by converting ammonia nitrogen into nitrate, thereby reducing ammonia levels and enhancing water quality (Spietz et al., 2015). Denitrifying bacteria, in contrast, convert nitrate into nitrogen gas, removing excess nitrogen from the water and preventing eutrophication (Holmes et al., 2019). However, an imbalance in the bacterial community, characterized by a decline in beneficial bacteria such as nitrifying and denitrifying bacteria and an increase in harmful bacteria, can negatively impact water quality. This imbalance may lead to elevated levels of ammonia nitrogen and nitrite, along with decreased dissolved oxygen (Fitzgerald et al., 2015; Guo et al., 2009). Such unfavorable conditions can impair the growth of *S. paramamosain*, compromise its immune system, and increase its susceptibility to diseases (Chen and Wang, 2019). To address this, particularly in the context of expanding aquaculture to northern regions, there is a critical need to fill the gaps in microbial data from saline-alkaline ponds in the north and analyze their ecological differences from seawater pond communities. This research will provide insights into how bacterial communities in these distinct environments influence water quality and, consequently, the health and growth of *S. paramamosain*. Therefore, studying the structure and diversity of bacterial communities in seawater and saline-alkaline ponds in northern regions, and their interactions with physicochemical water quality parameters, is essential for optimizing aquaculture conditions and ensuring the healthy growth of *S. paramamosain*.

This study focuses on a comparative analysis of bacterial communities in seawater and saline-alkali aquaculture ponds for *S. paramamosain* in northern China. Using 16S rRNA gene sequencing, we characterized the bacterial communities in both pond types and investigated their correlations with physicochemical water quality parameters. In northern China, seawater and saline-alkali ponds represent two dominant aquaculture environments, exhibiting marked differences in key parameters such as salinity, pH, and dissolved oxygen (DO). These differences may critically shape bacterial community structure and function. However, systematic comparisons of bacterial communities between these two environments remain limited, necessitating in-depth exploration of their interactions with aquaculture conditions. To address this gap, we analyzed the composition of bacterial communities in seawater and saline-alkali ponds and their interactions with environmental factors, aiming to elucidate the characteristics and dynamics of microbial communities across distinct aquaculture systems. Specifically, this study addresses the following questions: (1) how do bacterial community structure and diversity differ between seawater and saline-alkali ponds? (2) Which environmental factors predominantly drive these structural and functional differences? (3) Are specific bacterial taxa strongly associated with a particular pond type? (4) What functional differences exist in bacterial communities between the two environments? By addressing these questions, this work provides a scientific foundation for optimizing aquaculture environments for *S. paramamosain* in northern China, enhancing productivity and quality, and promoting sustainable development of regional aquaculture.

## 2 Materials and methods

### 2.1 Experimental settings and breeding management

From May 1 to September 30, 2022, a five-month breeding experiment on *S. paramamosain* was conducted in seawater ponds (118°58′E, 37°37’N) at Haiyue Aquatic Science and Technology Co., Ltd., Dongying City, and saline-alkali ponds (118°56′E, 37°40’N) at Zhengda Sangtian Agricultural Development Co., Ltd., Dongying City. Both pond types were rectangular, with a surface area of 2,240 m^2^ and a water depth of 1.2 m. Prior to the experiment, all ponds were disinfected using bleaching powder and enclosed with plastic film to prevent crab escape. Oxygen supplementation was provided as needed, based on weather conditions and the feeding and activity levels of the crabs.

Juvenile *S. paramamosain* crabs in the C4 stage, weighing 2 ± 0.2 g, were obtained from Ningbo Huada Haichang Co., Ltd., for experimental aquaculture. Only active, healthy juveniles with intact limbs were selected for the experiment. The stocking density in each pond was set at 1.2 crabs per square meter. Throughout the aquaculture period, one-third of the total water volume in each pond was replaced monthly. All crabs were fed once daily with artificial feed sourced from Ningbo Haiding Aquatic Feed Co., Ltd., at a feeding rate of 10–15%, which was consistent across all experimental groups. The formulated feed for *S. paramamosain* included fish meal, squid paste, soybean meal, shrimp meal, brewer’s yeast, phospholipids, refined fish oil, high-gluten flour, minerals, trace elements, vitamins, and polysaccharides.

### 2.2 Sample collection

In this study, water samples were collected on September 29, 2022, from three seawater ponds and three saline-alkali ponds. The sampling locations were positioned at the center of each pond, and water was collected at a depth of 0.6 m. At each sampling site, six 100 mL water samples were collected using pre-sterilized polycarbonate sampling bottles, resulting in a total of 36 water samples. To prevent potential microbial contamination, all sampling equipment was sterilized by high-temperature autoclaving before use. The collected water samples were thoroughly homogenized on-site and then aliquoted into sterile centrifuge tubes. The samples were immediately stored at 4°C to minimize microbial activity and chemical alterations. Upon completion of sampling, all water samples were promptly transported to the laboratory. Sample processing was conducted in a biosafety cabinet under sterile conditions, and all aliquoting procedures were completed within 2 h. The processed samples were then immediately stored in an ultra-low temperature freezer at −80°C for further analysis. All water samples were classified according to the alphanumeric identifiers provided in Table 1 and further subdivided as specified in Table 2 to ensure traceability and accuracy in subsequent analyses.

**Table 1:** Naming of water samples in seawater ponds and saline-alkali ponds.

Identifier	Sampling pond	Sampling location	Sample type	Sampling date	Sample quantity
A	No. 1 Marine Pond	Center of the Pond	Water Sample	Sep 29, 2022	6
B	No. 2 Marine Pond	Center of the Pond	Water Sample	Sep 29, 2022	6
C	No. 3 Marine Pond	Center of the Pond	Water Sample	Sep 29, 2022	6
D	No. 1 Saline-Alkali Pond	Center of the Pond	Water Sample	Sep 29, 2022	6
E	No. 2 Saline-Alkali Pond	Center of the Pond	Water Sample	Sep 29, 2022	6
F	No. 3 Saline-Alkali Pond	Center of the Pond	Water Sample	Sep 29, 2022	6

**Table 2:** Classification of samples from seawater ponds and saline-alkali ponds.

Group	Sample 1	Sample 2	Sample 3	Sample 4	Sample 5	Sample 6
A	S1a	S1b	S1c	S1d	S1e	S1f
B	S2a	S2b	S2c	S2d	S2e	S2f
C	S3a	S3b	S3c	S3d	S3e	S3f
D	SA1a	SA1b	SA1c	SA1d	SA1e	SA1f
E	SA2a	SA2b	SA2c	SA2d	SA2e	SA2f
F	SA3a	SA3b	SA3c	SA3d	SA3e	SA3f

### 2.3 Physicochemical indexes of the water samples

The temperature, salinity, pH, dissolved oxygen (DO), and ammonia (NH₄^+^-N) concentrations in the water samples were measured using a handheld multi-parameter instrument (YSI Pro 10,102,030, Yellow Springs Instrument, United States). Additionally, nitrate nitrogen (NO₃^−^-N) concentrations were determined using the “Water Quality—Determination of Nitrogen (Nitrite)—Spectrophotometric Method” in accordance with the Chinese standard GB 7493–87, the detailed procedure is as follows: Initially, sample pre-treatment is conducted. If the pH exceeds 11, phenolphthalein indicator should be added, and phosphoric acid titrated until the red color disappears, adjusting the pH to 1.8 ± 0.3. In cases where the sample contains suspended solids or coloration, aluminum hydroxide suspension should be added, followed by stirring and filtration. Subsequently, a series of standard nitrite nitrogen solutions (ranging from 0 to 0.05 mg/L) are prepared. After adding the coloring agent, absorbance is measured to construct a standard curve. Next, 50 mL of the sample is mixed with 1 mL of the coloring agent and left to stand for 20 min before measuring the absorbance at a wavelength of 540 nm using a 10 mm cuvette. The concentration is then calculated based on the standard curve. Interfering substances such as chloramines and chlorine can be eliminated by adjusting the pH or adding masking agents. Reagents used must be of analytical grade, and water should be nitrite-free distilled water obtained through double distillation. Coloring agents should be freshly prepared and stored away from light. Instrumentation requirements include a UV spectrophotometer, with glassware cleaned using hydrochloric acid. Quality control measures encompass blank tests, precision (with an RSD ≤ 0.07%), and spike recovery rates (92.7–96.7%). This method quantifies through the absorbance of azo dyes, necessitating strict adherence to standardized procedures to ensure accurate and reliable results.

### 2.4 DNA extraction and purification

DNA extraction was performed using the cetyltrimethylammonium bromide (CTAB) method, following the protocol provided by Beijing Novogene Bioinformatics Technology Co., Ltd. Initially, 1,000 μL of CTAB lysis buffer was added to a 2.0 mL EP tube, followed by the addition of lysozyme and the sample (Aboul-Maaty and Oraby, 2019). The mixture was incubated at 65°C with periodic gentle inversion to ensure complete sample lysis. After incubation, the lysate was centrifuged at 12,000 rpm for 10 min, and the supernatant was transferred to a clean tube. An equal volume of phenol (pH 8.0): chloroform: isoamyl alcohol (25:24:1, v/v/v) was added, mixed thoroughly by inversion, and centrifuged at 12,000 rpm for 10 min. The aqueous phase was collected and subjected to a second extraction with chloroform:isoamyl alcohol (24:1, v/v), followed by centrifugation under identical conditions. DNA was precipitated by adding isopropanol to the supernatant, gently mixing, and incubating at −20°C. The precipitate was collected by centrifugation at 12,000 rpm for 10 min. The DNA pellet was washed twice with 75% ethanol, air-dried on a sterile surface, and dissolved in nuclease-free water (ddH₂O). To ensure DNA quality, the concentration and purity of the extracted DNA were measured using a NanoDrop 2000 spectrophotometer (Thermo Fisher Scientific), with absorbance ratios of A260/280 between 1.8 and 2.0 considered acceptable. Additionally, DNA integrity was verified by electrophoresis on a 1% agarose gel stained with ethidium bromide. To remove residual RNA, 1 μL of RNase A was added, and the mixture was incubated at 37°C for 15 min. The purified DNA was stored at −20°C for further analysis.

### 2.5 16S rDNA gene amplification and Illumina high-throughput sequencing

The V4 region of the 16S rDNA gene was amplified using the primer pair 515F (5’-GTGCCAGCMGCCGCGGTAA-3′) and 806R (5’-GGACTACHVGGGTWTCTAAT-3′) to assess bacterial diversity (Caporaso et al., 2011). Each polymerase chain reaction (PCR) was performed in a 25 μL reaction volume containing 15 μL of Phusion High-Fidelity PCR Master Mix, 0.2 μM of each primer, and 10 ng of genomic DNA as the template. The thermal cycling conditions consisted of an initial denaturation at 98°C for 1 min, followed by 30 cycles of denaturation at 98°C for 10 s, annealing at 50°C for 30 s, and extension at 72°C for 30 s. A final extension step was performed at 72°C for 5 min. PCR products were purified using magnetic beads, after which the amplicons were quantified. The purified PCR products were pooled equimolarity to generate a sequencing library. The quality and quantity of the prepared library were assessed using a Qubit fluorometer and quantitative PCR (qPCR). The sequencing was then performed on an Illumina platform to obtain paired-end reads.

### 2.6 Illumina data processing

Raw sequencing reads were demultiplexed based on unique barcode and primer sequences assigned to each sample. Paired-end reads were merged using FLASH (version 1.2.11) to generate raw tags, followed by quality filtering with fastp (version 0.23.1) to obtain high-quality clean tags (Magoc and Salzberg, 2011; Bokulich et al., 2013). Chimeric sequences were identified and removed using the UCHIME algorithm, with reference-based filtering performed against the Silva database, resulting in effective tags (Edgar et al., 2011). Amplicon sequence variants (ASVs) were generated using the DADA2 plugin in QIIME2 (Maruyama et al., 2020). Taxonomic classification of ASVs was carried out with QIIME2, utilizing the Silva 138.1 database for bacterial sequences. Data normalization was performed using the sample with the lowest sequence count to ensure consistency across datasets. Subsequent analyses included alpha diversity metrics (e.g., Shannon and Simpson indices) and beta diversity evaluations [e.g., principal coordinates analysis (PCoA) and unweighted pair group method with arithmetic mean (UPGMA) clustering]. Taxonomic profiles, heatmaps, and diversity indices were visualized using R packages such as ggplot2 and pheatmap. These analyses provided a comprehensive overview of bacterial community composition and structure across samples.

### 2.7 IndVal method

The Indicator Value (IndVal) method is grounded in the concept that a species’ association with a specific habitat is determined by its frequency of occurrence and abundance within that habitat (Leote et al., 2020). This method assesses the relationship between species and habitats by calculating a composite index that integrates both the species’ average abundance and its occurrence frequency across sample plots. The specificity of a species is represented by its occurrence rate within a particular habitat, reflecting how exclusive the species is to that habitat. Fidelity, in contrast, refers to the proportion of the species’ mean abundance within the habitat, indicating its consistency and prevalence in the sampled environment. In the R programming environment, the indicspecies package is used to compute the IndVal index, where the package’s functions automatically calculate both specificity and fidelity based on species’ occurrence and abundance data from the sample plots. The resulting IndVal value quantifies the strength of the species’ association with a given habitat, with higher values indicating a stronger relationship. A higher IndVal score suggests that a species is more indicative of the habitat, thereby enhancing its potential as an indicator species for that habitat.

### 2.8 Statistical analysis

All statistical analyses were performed using IBM SPSS Statistics 20, with independent Student’s *t*-tests applied to assess significant differences. Alpha diversity indices, including Chao1, Pielou_e, Shannon, and Simpson indices, were calculated using MetaParallel v3.5.2 (Jing et al., 2017). Relative abundance maps for the top 10 phyla and genera were created using Microsoft Excel 2010. The UPGMA cluster tree for water samples collected from seawater and saline-alkali ponds was constructed using the ape, phangorn, and ggtree packages in R. Principal coordinate analysis (PCoA) and non-metric multidimensional scaling (NMDS) were conducted at the phylum and genus levels using Bray–Curtis distance matrices, implemented with the WGCNA package in R v3.6.0. Environmental variables correlated with bacterial community composition were identified using the BioEnv procedure in the vegan package in R v3.6.0 (Li X. et al., 2024; Li Z. et al., 2024). Canonical correspondence analysis (CCA) was performed using the vegan and ggplot2 packages in R v3.6.0 to examine the contribution of environmental factors to microbial community shifts. Indicator species associated with seawater and saline-alkali ponds were identified using the labdsv package in R. Pearson correlation analyses were conducted in R v3.6.0 to assess the relationships between indicator species and physicochemical water quality parameters. Finally, functional annotation of microbial communities was performed using PICRUSt2, and clustering heatmaps of functional genes and sample groups were generated using the pheatmap package, with KEGG pathway annotations retrieved via the KEGGREST package in R.

## 3 Results

### 3.1 Analysis of physical and chemical indexes of water samples in seawater ponds and saline-alkali ponds

A comparative analysis was performed on the physicochemical parameters of water quality between seawater ponds (Groups A, B, C) and saline-alkali ponds (Groups D, E, F) used for *S. paramamosain* aquaculture (Table 3). The water temperature in the seawater ponds (27.30–27.67°C) was slightly higher than in the saline-alkali ponds (26.50–27.67°C), though the difference was not statistically significant (*p* > 0.05). In contrast, salinity levels in the seawater ponds (27.40–27.67 ppt) were significantly higher than in the saline-alkali ponds (7.33–7.80 ppt), with a statistically significant difference observed (*p* < 0.05). The pH in the seawater ponds (7.62–7.64) was slightly lower than in the saline-alkali ponds (8.67–8.72), and this difference was statistically significant (*p* < 0.05). Dissolved oxygen concentrations in the seawater ponds (8.83–8.91 mg/L) were significantly higher than in the saline-alkali ponds (5.89–6.25 mg/L), with a statistically significant difference (*p* < 0.05). Ammonia nitrogen concentrations were lower in the seawater ponds (0.30–0.37 mg/L), while higher levels were observed in the saline-alkali ponds (0.53–0.58 mg/L), with the difference being statistically significant (*p* < 0.05). Finally, nitrite nitrogen concentrations were significantly lower in the seawater ponds (0.027–0.031 mg/L) compared to the saline-alkali ponds (0.045–0.048 mg/L), with a significant difference (*p* < 0.05).

**Table 3:** Analysis of physical and chemical indexes of water samples in seawater ponds and saline-alkali ponds.

Sample	Temperature (°C)	Salinity (ppt)	pH	Dissolved oxygen (mg/L)	Ammonia nitrogen (mg/L)	Nitrite nitrogen (mg/L)
A	27.67 ± 1.15^a^	27.67 ± 1.15^a^	7.63 ± 0.02^b^	8.83 ± 0.55^a^	0.37 ± 0.08^b^	0.031 ± 0.001^b^
B	27.30 ± 1.25^a^	27.60 ± 1.30^a^	7.64 ± 0.03^b^	8.91 ± 0.60^a^	0.35 ± 0.10^b^	0.028 ± 0.002^b^
C	26.50 ± 1.30^a^	27.40 ± 1.25^a^	7.62 ± 0.02^b^	8.86 ± 0.50^a^	0.30 ± 0.10^b^	0.027 ± 0.002^b^
D	27.67 ± 0.58^a^	7.33 ± 0.58^b^	8.68 ± 0.03^a^	6.25 ± 0.61^b^	0.58 ± 0.13^a^	0.048 ± 0.005^a^
E	26.80 ± 0.65^a^	7.80 ± 0.65^b^	8.72 ± 0.03^a^	6.17 ± 0.65^b^	0.55 ± 0.15^a^	0.046 ± 0.006^a^
F	27.10 ± 0.60^a^	7.60 ± 0.60^b^	8.67 ± 0.02^a^	5.89 ± 0.60^b^	0.53 ± 0.14^a^	0.045 ± 0.005^a^

Letters A, B, and C indicate water samples from seawater ponds, letters D, E, and F indicate water samples from saline-alkali ponds, and different superscript letters in the same column indicate significant differences between groups (*P* < 0.05). The same below.

### 3.2 Diversity analysis of bacterial communities in seawater pond and saline-alkaline pond

**Figure 1:**
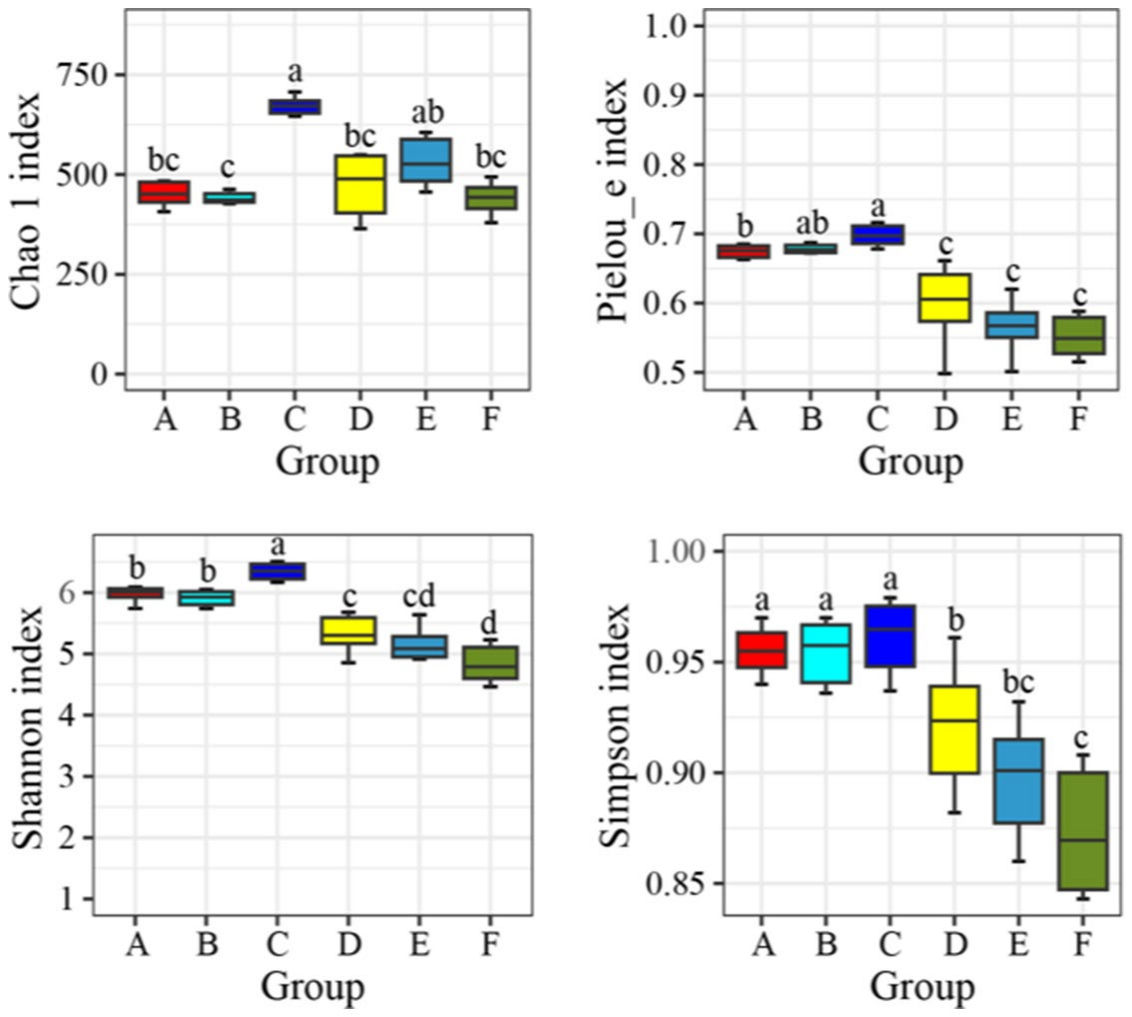
Diversity index of bacteria and microorganisms in seawater ponds and saline-alkali ponds. The data indicates the mean standard deviation, and different letters indicate significant differences between groups (*p* < 0.05).

A comparative analysis was performed on the microbial diversity indices of water samples from seawater ponds (Groups A, B, C) and saline-alkali ponds (Groups D, E, F). The indices included the Chao 1 index, Pielou_e evenness index, Shannon diversity index, and Simpson index (Figure 1). The Chao 1 index indicated that species richness was highest in Group C, significantly exceeding that of Groups A and B in the seawater ponds, as well as all groups in the saline-alkali ponds (*p* < 0.05). Among the saline-alkali ponds, Group D exhibited relatively high species richness, whereas Groups E and F had lower richness, with significant differences observed when compared to the seawater ponds (*p* < 0.05). Overall, species richness was higher in seawater ponds compared to saline-alkali ponds. The Pielou_e evenness index showed that the microbial community in Group C had the highest evenness, significantly higher than that in Groups D, E, and F (*p* < 0.05). No significant differences in evenness were observed among Groups A, B, and C in the seawater ponds (*p* > 0.05). Evenness was generally lower in the saline-alkali ponds, with Group F showing the lowest evenness, indicating a more uneven microbial community distribution. The Shannon diversity index revealed that Group C had the highest diversity, significantly surpassing that of all saline-alkali pond groups (*p* < 0.05). Groups A and B followed, while Groups D, E, and F exhibited progressively lower diversity, with Group F being the lowest. Microbial diversity in the saline-alkali ponds was notably lower than in the seawater ponds. The Simpson index further confirmed these findings. Group C had a Simpson index close to 1, suggesting low dominance of specific microbial species and a more balanced community structure. Groups A and B showed slightly lower Simpson indices. In contrast, Groups D, E, and F had even lower Simpson indices, with Group F being the lowest, indicating fewer dominant microbial species and a less balanced community structure.

### 3.3 Composition analysis of bacterial communities in seawater ponds and saline-alkali ponds

**Figure 2:**
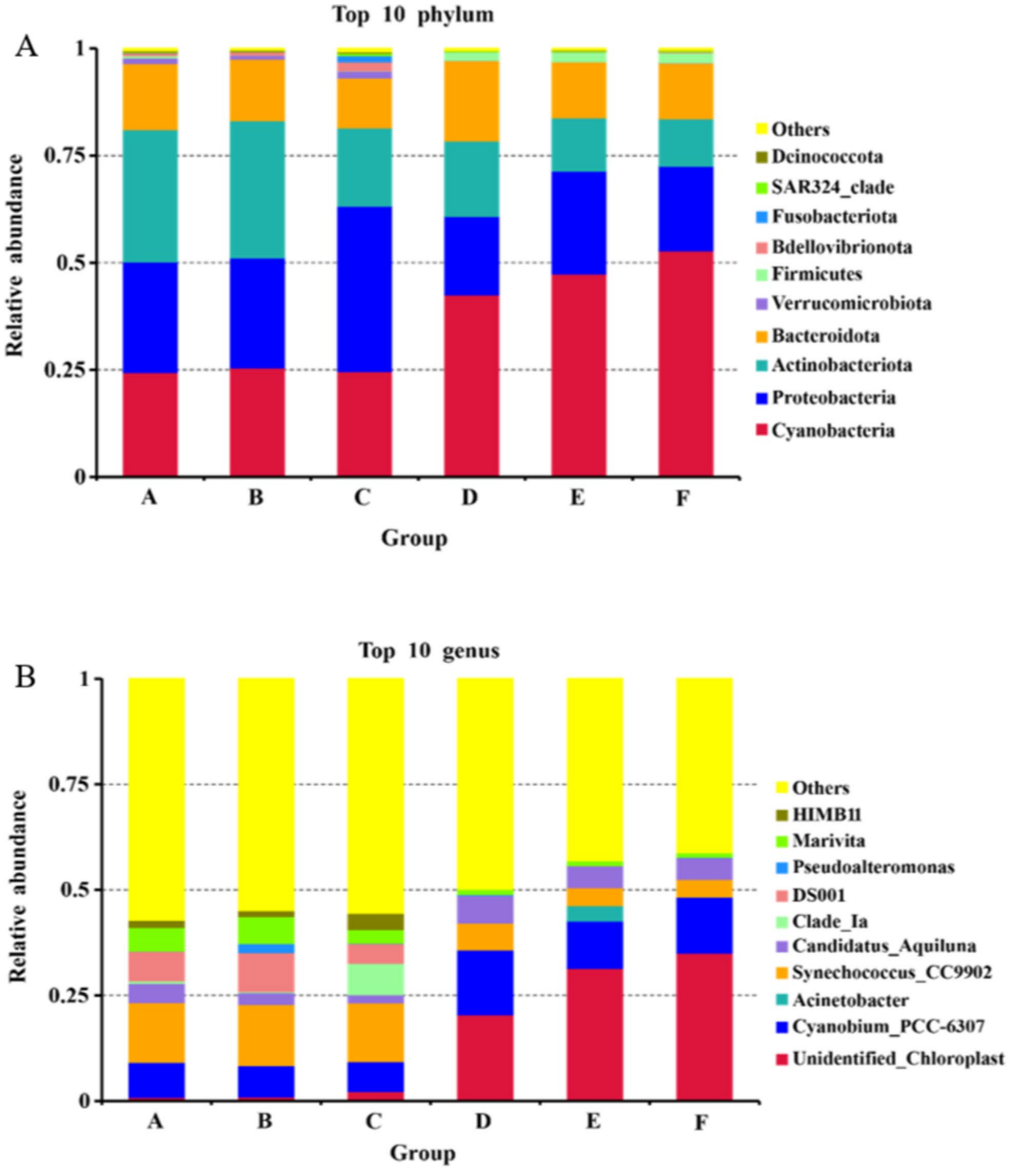
The relative abundance maps of the top 10 bacteria in seawater ponds and saline-alkali ponds are **(A)** at the level of phylum, and **(B)** at the level of genus.

At the phylum level, there was minimal variation in the dominant bacterial communities between the seawater and saline-alkali ponds, which were primarily composed of *Cyanobacteria*, *Proteobacteria*, *Actinobacteriota*, and *Bacteroidota* (Figure 2). However, the relative abundances of *Verrucomicrobiota* and *Bdellovibrionota* were significantly higher in the seawater ponds, while *Cyanobacteria* and *Firmicutes* were more abundant in the saline-alkali ponds. At the genus level, significant differences were observed in the relative abundances of bacterial communities between the two pond types. In the seawater ponds, the dominant genera included *Cyanobium_PCC-6307*, *Synechococcus_CC9902*, *DS001*, and *Marivita*. In contrast, the dominant genera in the saline-alkali ponds were *unidentified_Chloroplast*, *Cyanobium_PCC-6307*, *Synechococcus_CC9902*, and *Candidatus_Aquiluna*.

### 3.4 Structural analysis of bacterial communities in seawater ponds and saline-alkali ponds

**Figure 3:**
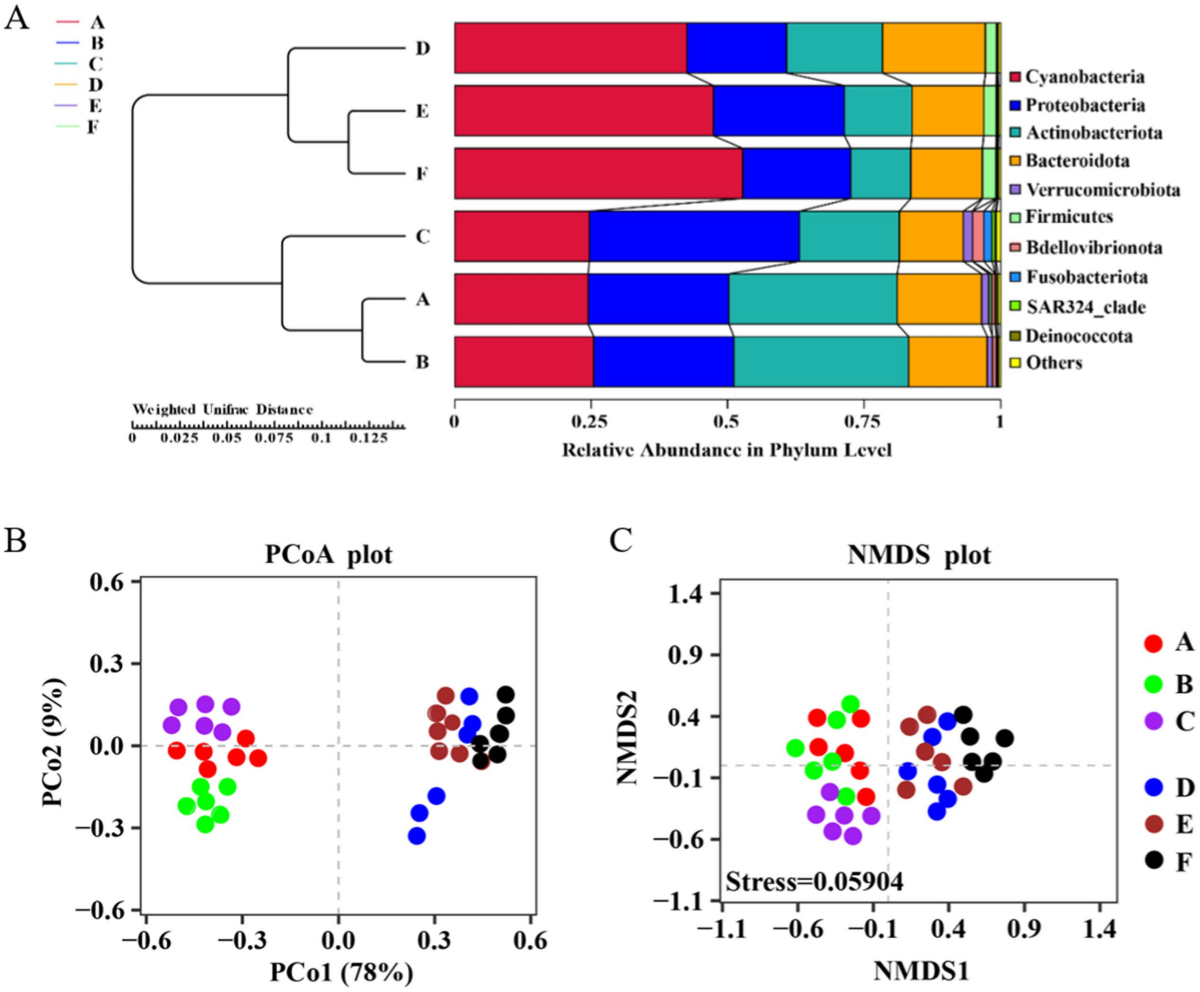
**(A)** UPGMA cluster tree of seawater pond and saline-alkali pond water samples, **(B)** PCoA map and **(C)** NMDS map of bacterial communities in the aquaculture ponds.

From Figure 3A, it is evident that Groups A, B, and C cluster together, as do Groups D, E, and F, forming two distinct branches. This clustering indicates a notable difference in the bacterial community structure at the phylum level between the seawater and saline-alkali ponds. In the seawater ponds, the bacterial communities are primarily dominated by *Cyanobacteria*, *Proteobacteria*, *Actinobacteriota*, and *Bacteroidetes*, with each phylum contributing approximately 25% to the overall relative abundance. The close clustering of samples from the seawater ponds suggests a high degree of similarity, indicating that the microbial community structure is stable and uniformly distributed across these ponds. In contrast, the bacterial community structure in the saline-alkali ponds shows greater variability. Dominant phyla in these ponds include *Cyanobacteria*, *Proteobacteria*, *Actinobacteriota*, and *Bacteroidetes*, along with smaller proportions of *Firmicutes* and other bacterial groups. Notably, *Cyanobacteria* accounts for a substantial 50% of the relative abundance in the saline-alkali ponds, reflecting a distinct microbial composition compared to the seawater ponds. When examining the clustering tree, the branch distances between groups within the seawater ponds are relatively short, which suggests that the microbial communities in these ponds are similar and tightly clustered. Similarly, groups within the saline-alkali ponds show short distances, indicating comparable microbial structures within these ponds. However, the distances between the seawater and saline-alkali pond groups are markedly larger, underscoring significant differences in the microbial community composition between the two pond types.

The PCoA plot (Figure 3B) illustrates the distribution of different samples in the principal coordinate space. The first principal coordinate (PCo1) accounts for 78% of the variation, while the second (PCo2) explains an additional 9%. The plot clearly reveals a distinct clustering pattern, with samples from Groups A, B, and C (seawater ponds) forming a cluster on the left side, and samples from Groups D, E, and F (saline-alkali ponds) grouping together on the right. This separation indicates significant differences in bacterial community structure between the seawater and saline-alkali ponds. The NMDS plot (Figure 3C) further supports this observation. With a stress value of 0.05904, the NMDS analysis demonstrates good reliability of the results. Similar to the PCoA plot, the NMDS plot shows a clear separation between the seawater pond and saline-alkali pond samples, with Groups A, B, and C clustering together, while Groups D, E, and F form a distinct cluster. This segregation highlights the significant differences in bacterial community composition between the two pond types, reinforcing the findings from the PCoA analysis.

### 3.5 Relationship between bacterial communities and environmental factors

**Figure 4:**
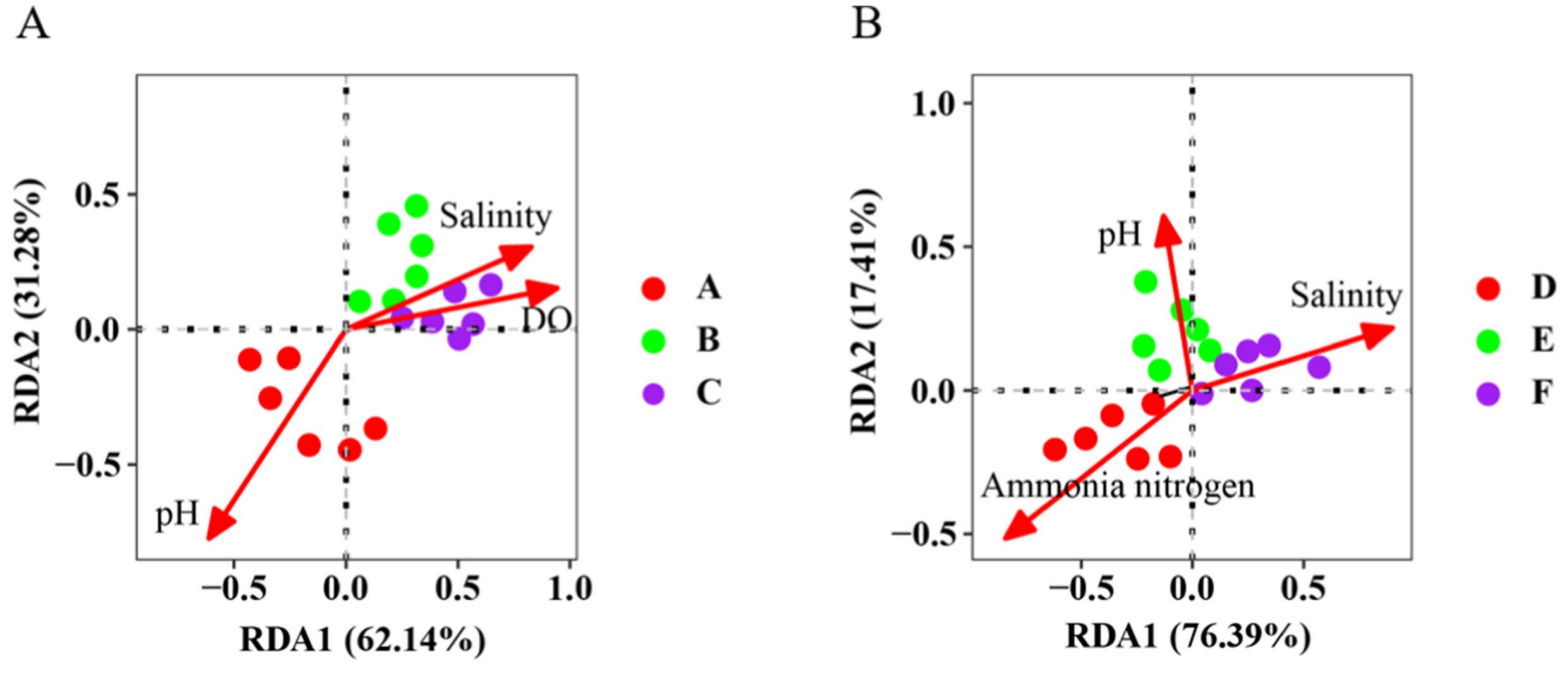
Bidirectional RDA of bacterial communities in **(A)** the Seawater pond water sample and **(B)** the Saline-alkali water pond water sample and their main environmental factors (DO, dissolved oxygen).

The environmental factors of water samples from the seawater ponds were standardized to eliminate dimensional differences. BioEnv analysis revealed that the most influential combination of environmental factors driving bacterial community variations was salinity, pH, and dissolved oxygen, with a correlation of r = 0.76. These factors were then used in the redundancy analysis (RDA). Salinity (positively correlated, *r* = 0.83), dissolved oxygen (positively correlated, *r* = 0.94), and pH (negatively correlated, *r* = −0.61) emerged as the key environmental factors along the first (RDA1) and second (RDA2) axes, collectively explaining 96.9% of the variation in the bacterial community (Figure 4A). Similarly, the environmental factors of water samples from the saline-alkali ponds were standardized to address dimensional differences. BioEnv analysis identified salinity, pH, and ammonia nitrogen as the most relevant factors (correlation, *r* = 0.83). These were subsequently used in RDA. Salinity (positively correlated, *r* = 0.91), pH (positively correlated, *r* = 0.61), and ammonia nitrogen (negatively correlated, *r* = −0.83) were the primary environmental factors along RDA1 and RDA2. Together, they explained 93.8% of the variation in the bacterial community (Figure 4B).

### 3.6 Correlation analysis between physical and chemical indexes of water samples and bacterial communities

The IndVal method was applied at the genus level to identify sensitive bacterial species, with an indicator value threshold set at 0.65 (*p* < 0.01). The distribution characteristics of these sensitive species are summarized in Table 4. Several sensitive bacterial species were identified in the seawater ponds. *Sphingoaurantiacus* and *Cobetia* were strongly associated with Group B, with indicator values of 0.6714 and 0.6647, respectively, and low probability values (0.004 and 0.003). This indicates a clear specific distribution tendency in seawater ponds. Additionally, *Propionigenium*, *unidentified_SAR116_clade*, *Clade_Ia*, and *unidentified_Clade_III* were associated with Group C, exhibiting high indicator values of 0.9504, 0.9083, 0.8979, and 0.8777, respectively, all with a probability value of 0.001. These results underscore their strong indicative role in seawater ponds.

**Table 4:** Distribution characteristics of indicator bacteria in seawater ponds and saline-alkali ponds.

Indicator species	Group	Pond	Indicator_value	Probability
Sphingoaurantiacus	B	Seawater pond	0.6714	0.004
Cobetia	B	Seawater pond	0.6647	0.003
Propionigenium	C	Seawater pond	0.9504	0.001
Unidentified_SAR116_clade	C	Seawater pond	0.9083	0.001
Clade_Ia	C	Seawater pond	0.8979	0.001
Unidentified_Clade_III	C	Seawater pond	0.8777	0.001
Roseivivax	D	Saline-alkali pond	0.6744	0.001
Tropicimonas	D	Saline-alkali pond	0.6667	0.001
Thiobacillus	D	Saline-alkali pond	0.6638	0.001
Staphylococcus	E	Saline-alkali pond	0.7895	0.001
Peptoniphilus	E	Saline-alkali pond	0.6733	0.004
Brevibacterium	F	Saline-alkali pond	0.6667	0.002
Francisella	F	Saline-alkali pond	0.6855	0.001

In the saline-alkali ponds, *Roseivivax*, *Tropicimonas*, and *Thiobacillus* were predominantly associated with Group D, exhibiting indicator values ranging from 0.6638 to 0.6744, with a probability of 0.001. These species are characteristic of saline-alkali ponds. *Staphylococcus* and *Peptoniphilus*, which were linked to Group E, showed indicator values of 0.7895 and 0.6733, with associated probabilities of 0.001 and 0.004, respectively. These values underscore the significant distribution patterns of these species within saline-alkali ponds. Additionally, *Brevibacterium* and *Francisella*, linked to Group F, displayed indicator values of 0.6667 and 0.6855, with probabilities of 0.002 and 0.001, confirming their role as sensitive bacterial species in saline-alkali ponds. These findings offer valuable insights into the distinct microbial community structures between seawater and saline-alkali ponds, forming a foundation for future ecological research and aquaculture applications.

**Figure 5:**
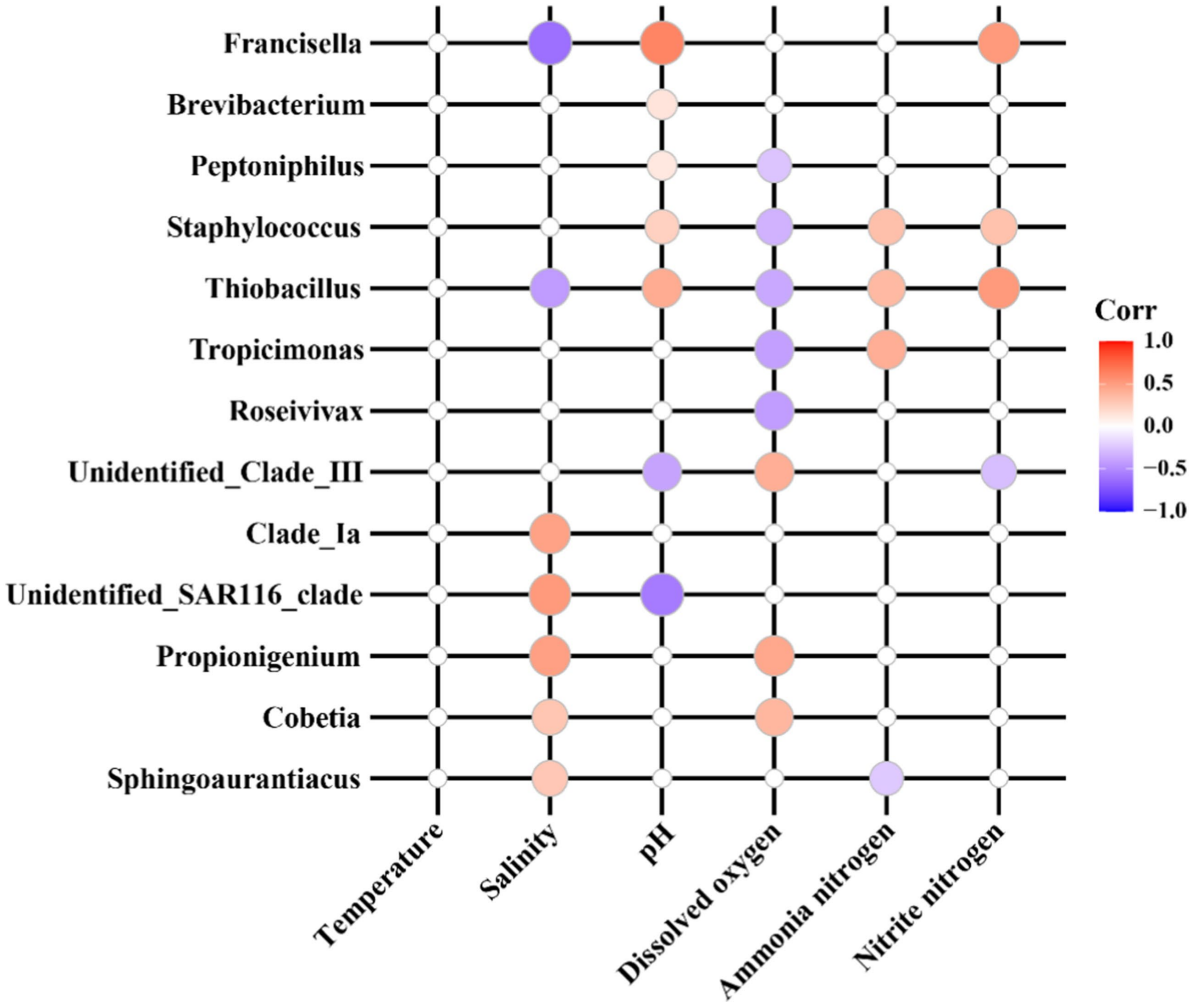
Correlation analysis between sensitive indicator flora and physical and chemical indexes of water quality.

The Pearson correlation heatmap (Figure 5) illustrates significant relationships between various sensitive bacterial species and water quality physicochemical parameters. The color intensity of the heatmap reflects the strength of these correlations, with red denoting positive correlations and blue indicating negative correlations. Specifically, salinity exhibited a positive correlation with *Clade_Ia*, *unidentified_SAR116_clade*, *Propionigenium*, *Cobetia*, and *Sphingoaurantiacus* in seawater pond samples, while it showed a negative correlation with *Francisella* and *Thiobacillus* in saline-alkali pond samples. pH demonstrated a negative correlation with *unidentified_Clade_III* and *unidentified_SAR116_clade* in seawater ponds, but a positive correlation with *Thiobacillus* and *Francisella* in saline-alkali ponds. Dissolved oxygen was positively correlated with *unidentified_Clade_III*, *Propionigenium*, and *Cobetia* in seawater ponds, whereas it was negatively correlated with *Roseivivax*, *Tropicimonas*, *Thiobacillus*, *Staphylococcus*, and *Peptoniphilus* in saline-alkali ponds. Ammonia nitrogen showed a positive correlation with *Tropicimonas*, *Thiobacillus*, and *Staphylococcus* in saline-alkali ponds, but a negative correlation with *Sphingoaurantiacus* in seawater ponds. Nitrite nitrogen was positively correlated with *Thiobacillus*, *Staphylococcus*, and *Francisella* in saline-alkali ponds, while it was negatively correlated with *unidentified_Clade_III* in seawater ponds.

### 3.7 Function prediction analysis

**Figure 6:**
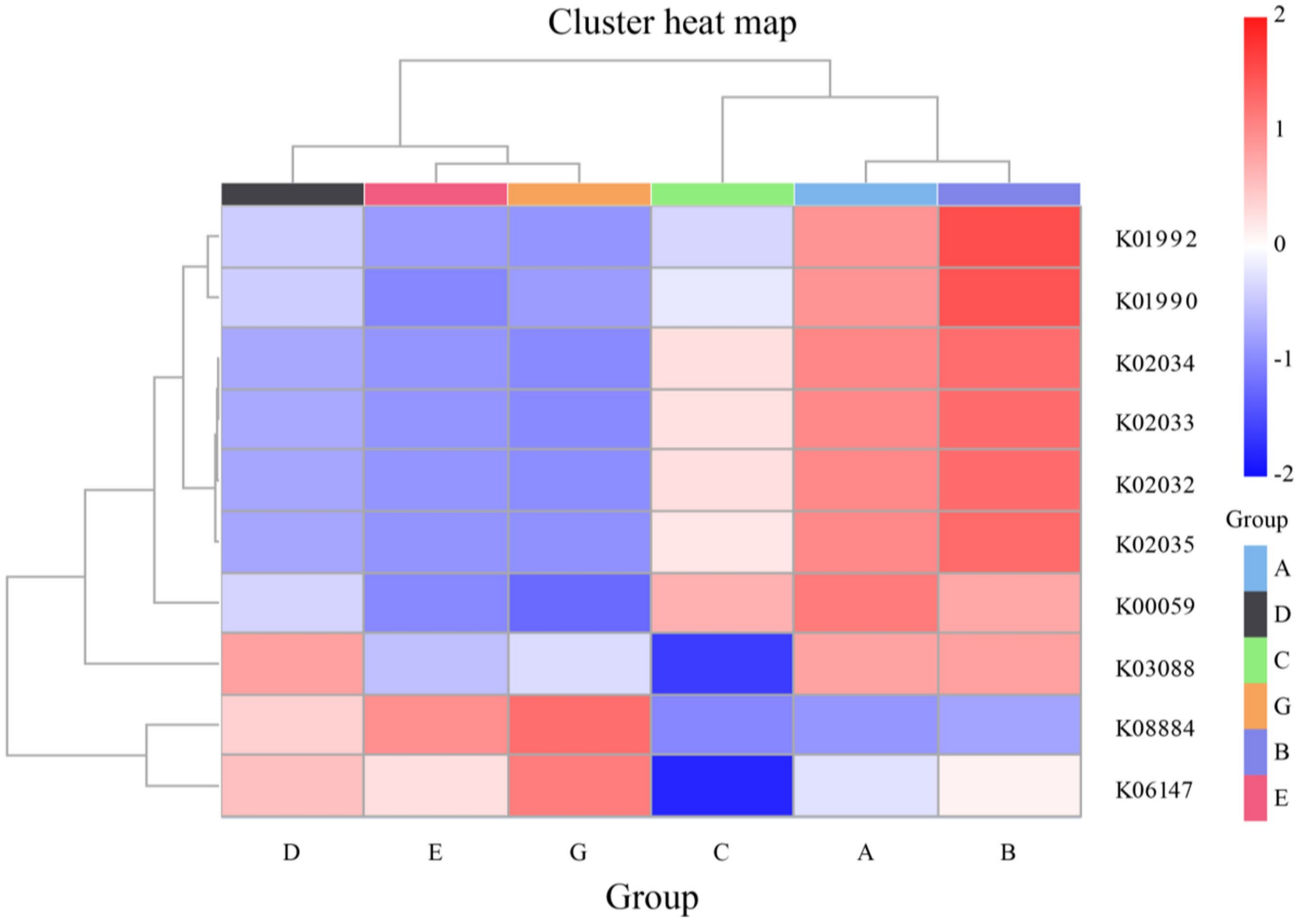
PICRUSt2 function annotation clustering heat map of seawater ponds and saline-alkali water ponds. The abscissa represents sample groups (D, E, G, C, A, B), and the ordinate displays KEGG orthologs (KOs) under positive selection (e.g., K01992, K01990). The color depth represents the relative abundance of functional genes.

Using PICRUSt2, functional gene annotation and clustering analyses were performed on water samples from seawater and saline-alkali ponds, revealing substantial differences in the functional composition of microbial communities between the two environments. The functional gene data for this analysis were sourced from the KEGG Orthology (KO) database. The clustering heatmap results demonstrated environmental specificity in the distribution of functional genes across the pond water samples, reflecting microbial adaptive strategies to their respective ecosystems (Figure 6). In saline-alkali ponds, microbial functional genes identified from the KEGG Orthology (KO) database were predominantly associated with resource acquisition and adaptation to environmental stress. For instance, genes K01992 and K01990, which encode sugar and glutamate transport proteins, were more abundant in saline-alkali environments. This suggests that microbes in these ponds actively acquire sugars and amino acids to cope with nutrient scarcity and high osmotic pressure. Moreover, the high prevalence of phosphate transport protein genes, such as K02034, K02033, and K02032, indicates that phosphorus availability in saline-alkali ponds may be limited, prompting microbes to enhance phosphorus utilization through high-affinity phosphate transport systems. In contrast, the functional genes in seawater ponds, also sourced from the KEGG Orthology (KO) database, were primarily related to nitrogen metabolism and protein synthesis. Genes K00059 and K06147, which are associated with glutamate dehydrogenase and nitrate reductase, respectively, exhibited high nitrogen cycling activity. The presence of gene K03088, encoding ribosomal proteins, suggests an increased capacity for protein synthesis, potentially to meet the higher nutrient inputs and dynamic environmental conditions in seawater ponds. Additionally, the gene K08884 (ATP-binding cassette transport protein), identified through the KEGG Orthology (KO) database, was significantly abundant in both environments, indicating that active resource transport via ABC transport systems is a common metabolic strategy among microbes. In summary, the functional differences in microbial community composition between seawater and saline-alkali ponds reflect the impact of environmental factors on gene distribution, as demonstrated by KEGG Orthology (KO) database analyses. Microbes in saline-alkali ponds are adapted to stress through efficient resource utilization and osmotic pressure regulation, while those in seawater ponds primarily engage in nutrient cycling and protein metabolism. These findings underscore the ecological significance of microbial functional diversity in these two distinct aquatic ecosystems.

## 4 Discussion

### 4.1 Diversity and structural differences of bacterial communities

The bacterial community diversity in seawater and saline-alkali ponds exhibits both similarities and significant differences. The Shannon and Simpson diversity indices reveal that while both pond types host a wide array of bacterial taxa, the diversity in the saline-alkali pond is marginally lower than in the seawater pond. This observation is consistent with studies by Liu et al. (2023) and others, who noted that extreme environments, such as saline-alkaline waters, tend to favor more specialized microbial communities (Niu et al., 2023). The dominance of halophilic and alkaliphilic bacteria in the saline-alkali pond further supports this notion (Valenzuela-Encinas et al., 2009). Contributing factors to the differences in bacterial diversity include variations in salinity, pH, and alkalinity, which exert selective pressure on microbial communities (Sorokin et al., 2012). Moreover, the more stable environmental conditions in seawater ponds, in contrast to the fluctuating conditions in saline-alkali ponds, may explain the higher diversity observed in the former (Paul and Mormile, 2017).

At the phylum level, both seawater and saline-alkali ponds are predominantly dominated by Cyanobacteria, Proteobacteria, and Actinobacteria, although their relative abundances differ significantly across the two environments. Seawater ponds exhibit a higher abundance of Proteobacteria, particularly the class Gammaproteobacteria, which aligns with the findings of Glaring et al. (2015) and reflects their adaptability to saline conditions. In contrast, Cyanobacteria and Firmicutes are more abundant in saline-alkali ponds, consistent with their reported tolerance to high alkalinity (Valenzuela-Encinas et al., 2009). At the genus level, Synechococcus_CC9902, DS001, and Marivita are highly abundant in seawater ponds, reflecting their salt tolerance and prevalence in marine environments (Ding et al., 2021). In contrast, unidentified_Chloroplast and Cyanobium_PCC-6307 dominate in saline-alkali ponds, indicating adaptation to high pH and ammonia nitrogen (Li X. et al., 2024; Li Z. et al., 2024). This finding is consistent with previous research by Kono and Spalding (2020), who observed alkaliphilic bacteria in high-pH environments.

The bacterial community structure was assessed using Principal Coordinates Analysis (PCoA), Non-metric Multidimensional Scaling (NMDS), and hierarchical clustering, revealing significant differences between the microbial communities of seawater and saline-alkali ponds. The seawater pond community exhibits a more even species distribution, consistent with Lyons and Schneider (1990), who observed balanced structures in stable, high-salinity environments. In contrast, saline-alkali ponds show pronounced clustering, indicating lower diversity but greater specialization. This pattern aligns with β-diversity indices and Kalwasińska et al. (2017), who linked extreme conditions to homogeneous communities dominated by specialized taxa. The observed differences in microbial community structure between the two pond types may be attributed to the unique combination of high alkalinity and pH in saline-alkali ponds, which impose stronger selective pressures on bacterial species (Yu et al., 2024; Sorokin et al., 2012).

### 4.2 Effects of water quality parameters on bacterial communities

This study reveals significant differences in the physicochemical water quality parameters between seawater and saline-alkali ponds, which have important implications for bacterial community structure. In high-salinity seawater ponds, bacterial community structure is relatively stable and exhibits higher diversity, with dominant groups like Cyanobacteria and Proteobacteria being evenly distributed. This is because salinity affects the osmotic pressure balance in bacterial cells, and high salinity selects for bacteria adapted to hyperosmotic environments, allowing them to dominate the community and influence its composition and diversity (Zahran, 1997). In contrast, the lower salinity in saline-alkali ponds leads to changes in microbial community structure, such as an increase in the relative abundance of Firmicutes, which may reflect adaptive strategies to lower salt concentrations (Sun et al., 2024).

pH, another critical factor, significantly impacts bacterial community composition. Seawater ponds, with lower pH (7.62–7.64), favor the dominance of Cyanobacteria and Proteobacteria, which play crucial roles in nutrient cycling (Landsman, 2019). Saline-alkali ponds, with higher pH (8.67–8.72), promote the proliferation of Cyanobacteria and Firmicutes, as these groups are better adapted to alkaline conditions (Belkin et al., 2024). The variation in pH alters bacterial metabolic activity and enzyme function, thereby affecting both community structure and functional diversity (Tu et al., 2022).

Dissolved oxygen (DO) concentrations differ markedly between the two pond types. Seawater ponds, with higher DO (8.83–8.91 mg/L), support aerobic bacteria and facilitate nitrogen cycling (Oren and Shilo, 1985), maintaining microbial stability and diversity. Saline-alkali ponds, with lower DO (5.89–6.25 mg/L), may promote anaerobic or facultative anaerobic bacteria, altering organic matter decomposition and nutrient cycling efficiency. These shifts are driven by differences in oxygen utilization and ecological niche distribution (Zhang et al., 2023; Pan et al., 2023).

### 4.3 Indicator bacteria and aquaculture environment stability

The differences in indicator bacteria between seawater and saline-alkali ponds can largely be attributed to the distinct environmental conditions in each ecosystem (Brauner et al., 2012; Heenatigala and Fernando, 2016), particularly variations in salinity, pH, and alkalinity. In seawater ponds, stable salinity and slightly alkaline pH favor indicator bacteria such as *Sphingoaurantiacus* and *Cobetia*, which are adapted to marine environments and sensitive to salinity fluctuations (Fernández-Juárez et al., 2022). In saline-alkali ponds, extreme alkalinity and pH select for alkaliphilic/halophilic species like *Brevibacterium* and *Thiobacillus*, reflecting adaptation to harsh conditions (Alvarado-López et al., 2023; Zhao et al., 2021).

Correlation analyses reveal contrasting relationships with physicochemical parameters. In seawater ponds, *Sphingoaurantiacus* and *Cobetia* positively correlate with salinity (Mu et al., 2020; Ganesan et al., 2023), indicating their role as salinity indicators. In saline-alkali ponds, *Brevibacterium* and *Thiobacillus* positively correlate with alkalinity/pH (Pei et al., 2021), while *Roseivivax* and *Thiobacillus* negatively correlate with dissolved oxygen and organic matter. This reflects adaptation to low-oxygen, nutrient-poor conditions (Cheng et al., 2024), aligning with previous studies on saline-alkaline environments (Ahmad et al., 2024).

*Sphingoaurantiacus* in seawater ponds negatively correlates with organic matter, likely due to competitive exclusion in nutrient-rich systems (Leite et al., 2023). These findings highlight the specificity of indicator bacteria as environmental health markers, underscoring the need to monitor salinity, pH, and dissolved oxygen to maintain microbial stability in *S. paramamosain* aquaculture.

### 4.4 The relationship between the prediction of bacterial community function and the culture of *S. paramamosain*

Functional prediction analysis reveals significant differences in the functional composition of microbial communities between seawater and saline-alkali ponds, highlighting the impact of environmental factors on the distribution of functional genes and microbial adaptation strategies. In saline-alkali ponds, microorganisms prioritize resource acquisition and stress resistance, as indicated by genes encoding glucose/glutamate transporters (K01992 and K01990) and high-affinity phosphate transport systems (K02034, K02033, and K02032), reflecting adaptive strategies to optimize phosphorus and nutrient utilization under limited availability (Knappe et al., 2003). In seawater ponds, microbial functions are dominated by nitrogen metabolism and protein synthesis, with genes such as glutamate dehydrogenase (K00059) and nitrate reductase (K06147), aligning with efficient nitrogen processing in nutrient-rich environments (Ahmed et al., 2021). Notably, ABC transporter genes (K08884) are highly expressed in both environments, indicating conserved strategies for active resource uptake. These functional differences suggest saline-alkali pond microbes rely on phosphorus optimization and osmotic regulation, while seawater pond microbes focus on nitrogen cycling and protein synthesis. For *S. paramamosain* aquaculture, these findings provide actionable insights: enhancing nitrogen metabolism in seawater ponds via optimized feed formulation and introducing phosphate-solubilizing microbes in saline-alkali ponds to leverage K02034/K02032 functions, thereby improving nutrient utilization and crab growth.

### 4.5 Limitations and future directions

This study has several limitations that warrant consideration. First, the reliance on a single sampling event (September 2022) may overlook seasonal variations in microbial dynamics, necessitating multi-seasonal sampling to comprehensively assess temporal shifts in bacterial communities. Second, while PICRUSt2 provided functional predictions of microbial metabolism, experimental validation through metatranscriptomic or enzymatic assays is critical to confirm the expression and activity of identified genes. Third, proposed management strategies, such as probiotic supplementation or phosphorus optimization, require field trials to evaluate their practical efficacy in enhancing *S. paramamosain* survival and growth under real-world aquaculture conditions. Addressing these limitations will strengthen the robustness of findings and facilitate actionable insights. Furthermore, indicator species in seawater and saline-alkali ponds, such as *Sphingoaurantiacus*, *Cobetia*, *Roseivivax*, *Tropicimonas*, and *Thiobacillus* etc., could be harnessed for synergistic regulation of harmful substances in water bodies through multiple mechanisms, which represents a promising future research direction. Future research should integrate multi-omics approaches (e.g., metagenomics and host-microbiome interaction studies), environmental modulation, and controlled aquaculture trials to optimize microbial management strategies. Such efforts will advance sustainable practices for *S. paramamosain* farming in northern China’s saline-alkali ecosystems, balancing productivity with ecological resilience.

## Data Availability

The datasets presented in this study can be found in online repositories. The names of the repository/repositories and accession number(s) can be found: https://www.ncbi.nlm.nih.gov/, PRJNA1232404.
